# General practitioners' reasoning when considering the diagnosis heart failure: a think-aloud study

**DOI:** 10.1186/1471-2296-6-4

**Published:** 2005-01-15

**Authors:** Ylva Skånér, Lars Backlund, Henry Montgomery, Johan Bring, Lars-Erik Strender

**Affiliations:** 1 Department of Clinical Sciences, Center of Family Medicine (CeFAM), Karolinska Institutet, Stockholm, Sweden; 2 Department of Psychology, Stockholm University, Sweden; 3 Statisticon AB, Uppsala, Sweden

## Abstract

**Background:**

Diagnosing chronic heart failure is difficult, especially in mild cases or early in the course of the disease, and guidelines are not easily implemented in everyday practice. The aim of this study was to investigate general practitioners' diagnostic reasoning about patients with suspected chronic heart failure in comparison with recommendations in European guidelines.

**Methods:**

Think-aloud technique was used. Fifteen general practitioners reasoned about six case vignettes, representing authentic patients with suspected chronic heart failure. Information about each case was added successively in five steps. The general practitioners said their thoughts aloud while reasoning about the probability of the patient having chronic heart failure, and tried to decide about the diagnosis. Arguments for and against chronic heart failure were analysed and compared to recommendations in guidelines.

**Results:**

Information about ejection fraction was the most frequent diagnostic argument, followed by information about cardiac enlargement or pulmonary congestion on chest X-ray. However, in a third of the judgement situations, no information about echocardiography was utilized in the general practitioners' diagnostic reasoning. Only three of the 15 doctors used information about a normal electrocardiography as an argument against chronic heart failure. Information about other cardio-vascular diseases was frequently used as a diagnostic argument.

**Conclusions:**

The clinical information was not utilized to the extent recommended in guidelines. Some implications of our study are that 1) general practitioners need more information about how to utilize echocardiography when diagnosing chronic heart failure, 2) guidelines ought to give more importance to information about other cardio-vascular diseases in the diagnostic reasoning, and 3) guidelines ought to treat the topic of diastolic heart failure in a clearer way.

## Background

Chronic heart failure (CHF) is a major cause of morbidity and mortality and it has a considerable impact on the health care system [1]. In a recent study, the prevalence in Sweden was estimated at 1.3–2.5% [2]. Early detection of CHF has become increasingly important, as modern drug treatment has the potential to improve symptoms and quality of life, slow down the rate of disease progression, and improve survival. However, diagnosing CHF is known to be difficult, especially in mild cases, as many features of the condition are not organ specific, and there may be few clinical features in the early stages of the disease [3-5]. Most of the patients are old, which also makes the diagnosis difficult. Older patients may have atypical symptoms, they may suffer from other diseases, and they may be on treatment that modifies their symptoms [3]. Diagnosing CHF has been found to be especially difficult in women and in obese patients [4]. A large proportion of patients with CHF are managed by general practitioners (GPs), especially older patients and patients early in the course of disease, i.e. those patients for whom the diagnostic process is characterized by the greatest uncertainty [6].

The European Society of Cardiology adopted guidelines for diagnosing CHF in 1995, and these were revised in 2001 [7-9]. Swedish guidelines, based on the 1995 European guidelines, were published in 1996 by the Swedish Medical Products Agency [10]. However, guidelines are often not easily or accurately integrated into daily practice [11,12].

The full versions of the above-mentioned guidelines are comprehensive documents, covering epidemiology, aetiology, pathophysiology and diagnostic methods, but may be difficult to apply to specific diagnostic situations [13]. However, the recommendations are summarized in 1) a definition, 2) an algorithm for the diagnosis of CHF, and 3) a table of assessments to be performed routinely to establish the presence of CHF. The definition includes three criteria: *a*) one or more typical symptoms (at rest or during exercise), *b*) objective evidence of cardiac dysfunction (at rest), and *c*) response to treatment directed towards CHF (in cases where diagnosis is in doubt). Criteria *a *and *b *should be fulfilled in all cases. Echocardiography (ECHO) is mentioned as the single most effective tool in widespread clinical use for objective assessment of cardiac dysfunction. In the algorithm for the diagnosis of CHF, a sequence of investigations is recommended: suspect CHF because of symptoms and signs; assess presence of cardiac disease by electrocardiography, X-ray or Natriuretic peptides (where available); imaging by echocardiography; assess aetiology, degree, precipitating factors and type of cardiac dysfunction; additional diagnostic tests where appropriate; choose therapy. Table 1 shows the assessments to be performed routinely [9]. In the present study, the list of assessments recommended in Table 1 was used for evaluation of the GPs' diagnostic reasoning. For most Swedish GPs, the main source of knowledge regarding CHF diagnostics is probably locally adapted protocols developed by cardiologists, or by cardiologists and GP representatives in collaboration.

**Table 1:** Diagnostic assessments according to guidelines Assessments to be performed routinely to establish the presence and likely cause of heart failure (Eur Soc Cardiol 2001).

Assessments	The diagnosis of heart failure			Suggests alterantive or additional diagnosis
	Necessary	Supports	Opposes	
Appropriate symptoms	+++		+++ (if absent)	
Appropriate signs		+++	+ (if absent)	
Cardiac dysfunctioning on imaging (usually echocardiography)	+++		+++ (if absent)	
Response of symptoms or signs to therapy		+++	+++ (if absent)	
Electrocardiography			+++ (if normal)	
Chest X-ray		+ (if pulmonary congestion *or *cardiomegaly)	+ (if normal)	Pulmonary disease
Full blood count*				Anemia/secondary polycythemia
Biochemistry and urinalysis*				Renal or hepatic disease/diabetes
Plasma concentration of natriuretic peptides in untreated patients (where available)*		+ (if elevated)	+++ (if normal)	

+ = of some importance; +++ = of great importance

*) Recommended assessments which are *not *included in the corresponding table in the Medical Products Agency version of guidelines from 1996.

Relatively few studies on how patients suspected of having CHF are diagnosed have been performed in primary health care settings, and most of them report over-diagnosis [3,4,14-16]. In the present study we used written case vignettes (case descriptions) and think-aloud technique to study how GPs' diagnostic reasoning and diagnostic judgements about patients with suspected CHF are related to the recommendations in the European guidelines [9]. What clinical information is considered important by the GPs in the sense that it is used as an argument for or against the diagnosis of CHF? What information that is considered important for diagnosing CHF in the guidelines is also considered important by the GPs?

## Methods

### Think-aloud method

Process-tracing techniques are used to study the cognitive processes involved in decision-making such as, for example, how judgements change over time as new information is presented, and which decision rules are used [17]. A method that is often used to describe the sequence of thoughts behind decision-making is the think-aloud technique [18]. Subjects are instructed to say their thoughts aloud while performing a task, and the verbal reports are usually audio-taped, transcribed to a written form, and then analysed. The think-aloud technique has been used in a number of studies in the field of medical decision-making [13,19].

The value of conclusions reached in such studies depends on the validity of the think-aloud method, and on the reliability of the coding process. Thinking aloud while performing a task often lengthens the time for completing the task, but does not seem to change the accuracy of task fulfilment or the cognitive processes [18]. In a recent study we found that think-aloud data were at least as valid as ratings in describing a clinical decision process [20].

### Participants

All health care centres in northern Stockholm within a distance of 20–30 km from the city centre (*n *= 61) were listed and contacted in a random order. The distance from central Stockholm was chosen for practical reasons. In each health care centre the GPs were contacted in a random order by one of the authors (YS). Only one GP at each centre was included in the study, and this person had to be a specialist in family medicine. We contacted the GPs during their regular telephone hour, during the period October 2001 to October 2002. Our goal was to include 15 GPs in the study.

A total of 30 GPs were reached, and 15 agreed to participate. Those who declined to participate were not asked why they did so, but the majority of those who spontaneously gave a reason mentioned a heavy workload. The participants had been specialists in family medicine for an average of 14.8 (range 3–25) years, they were on average 52.7 (range 42–62) years of age, and six of them were men. The non-participating GPs were on average 52.7 (range 35–62) years of age, and seven of them were men.

### Case vignettes

Six case vignettes (CV), based on authentic patients, were presented to the participants. The information presented in the case vignettes was obtained from the patient records and included information about relevant diseases (e.g. coronary heart disease, stroke, diabetes), lifestyle factors (e.g. smoking, alcohol consumption), symptoms, signs, electrocardiography (ECG), chest X-ray findings, and ECHO. Chest X-ray and ECHO results were presented in the same format as in the patient records. ECHO results could contain information about ejection fraction (EF), valvular disorders and ventricular wall motility. The diagnoses made by the attending cardiologists (based on all available clinical information, including ECHO) were used as a reference standard when assessing the participants' diagnostic accomplishments.

The six cases were selected to represent patients with various types of potential diagnostic problems: A "prototypical" CHF patient (CV2), a patient with both CHF and chronic obstructive pulmonary disease (COPD) (CV6), a patient with CHF, tachycardia and mitral valve insufficiency (CV3), an obese non-CHF patient with normal ECG and EF (CV5), a non-CHF patient with COPD (CV4) and a non-CHF patient with alcohol abuse and a metabolic syndrome (CV1). Additional file 1 shows some of the characteristics of the six cases.

For one of the cases (CV3) there was a disagreement between the diagnosis according to the cardiologists and the diagnosis that could be deduced from a simplistic interpretation of the guidelines. This patient had typical clinical findings including gallop rhythm, cardiomegaly, and pulmonary congestion, but normal left ventricular function according to ECHO. It could therefore be categorized as not CHF according to the definition given in the guidelines. However, this patient also had a mitral valve insufficiency, which can give a "false normal" ejection fraction value: the left ventricle is emptied both forward (cardiac output) and backward (leakage through the mitral valve).

### Procedure

Before the sessions the GPs had received written information about the aim of the study (to study clinical judgements) and about the method (think aloud), but not about the kind of medical problems that would be presented to them. The study was conducted at the GPs' offices. All visits and recordings were made by one of the authors (YS). The participants were instructed that six authentic patients, suspected by GPs to have CHF, would be presented, and that their task was to say aloud their thoughts about the case, and to try to decide whether the patient had CHF or not.

The order of the cases was the same for all participants. The order in which the information was presented was arranged to be as realistic as possible in relation to clinical practice (first history and symptoms, then findings, and then results of investigations). Each vignette was presented on a computer screen in five successive steps using QA software [21]. All previously shown information about a case was repeated at the top of the later screens in a different colour to reduce and control for memory effects. The participants could control the shift to a new screen by clicking with the mouse on a continue button at the bottom of the screen. After all the information had been presented, the participants were asked, on the sixth screen, to summarize their judgements about the case and to try to decide about the diagnosis. The doctors could express their diagnostic judgements freely, with their own words.

The doctors first got a test case (not recorded) in order to get acquainted with the think-aloud method, and then continued with the six study cases. The only intervention from the researcher during the think-aloud session was that a participant who was silent for more than about 15 seconds was reminded to say his or her thoughts aloud about the information presented [18]. All sessions were recorded and transcribed by a secretary.

### Response measures and coding of data

#### Coding of variables in the case vignettes

All information in the case vignettes that was of relevance for the diagnosis and that could take on different values was considered to be variables. Fifty variables were defined: 19 of them were included in all six vignettes (e.g. symptoms, signs and investigations mentioned in the guidelines), six in five, one in four, six in three, one in two, and 17 in one vignette (e.g. alcohol abuse, history of a bypass operation, and panic disorder). For each case vignette, the presented variables were coded for content and value (Table 2).

**Table 2:** Variabel codings

Information as presented in the case vignette	Content	Value
"Shortness of breath on level"	Dyspnoea	Positive (presence of finding)
"Pathological R-progression on ECG"	ECG	Positive (pathology)
"He has not had swollen legs"	Oedema	Negative (absence of finding)
"Regular rhythm"	Rhythm	Negative (normality)
"Relative heart volume 630 ml/m^2^"	Heart volume	630 (numeric values as presented in the text)

Examples of coding of variables in the case vignettes. Positive value = precense of finding, or pathological finding, negative value = absence of finding, or normal finding.

#### Coding of think-aloud protocols

For each participant, every mention of a variable was coded for how the GP seemed to use it: as an argument for the diagnosis of CHF, as an argument against CHF, or as not being of any explicit use for the diagnosis (mentioned only). "He has basal rales. This guy has CHF!" is an example of a participant using the variable "rales" (positive value) as an argument for CHF. "So I'm not really sure that he has got CHF. Just a moderate cardiac enlargement, no, I wouldn't think so" is an example of a participant using the variable "relative heart volume" (value 630 ml/m^2^) as an argument against CHF. For each participant, a specific evaluation of each variable value was only counted once for each case vignette in order not to give more weight to thoughtful repetitions of an argument than to a single, firm statement. However, if a participant used the same variable value as an argument both for and against the diagnosis of CHF, both evaluations were coded. Ten percent of the 90 case vignette protocols were selected at random and coded independently by two of the authors (YS, LB) to estimate the interrater agreement of the coding process. The rest of the protocols were coded by one of the authors (YS).

#### Comparing think-aloud protocols with guidelines

The list of diagnostic assessments recommended in the guidelines (Table 1) was used for comparing GPs' diagnostic reasoning with the guidelines. Breathlessness, ankle swelling, and fatigue are mentioned in the guidelines as appropriate symptoms, and leg oedema, tachycardia, gallop rhythm, and pulmonary crepitations (rales) as appropriate signs. (Neck vein distension and liver enlargement are also mentioned, but these signs were not present in the case vignettes.) Use of the variables in relation to recommendations was analyzed for frequency among GPs and case vignettes.

#### Classification of diagnostic judgements

The participants were not forced to express their diagnostic judgements in a specific format, and their free verbal statements therefore had to be interpreted and coded. Two of the authors (YS, LB) independently classified all the diagnostic judgements (*n *= 90) in three categories: CHF or probably CHF; uncertainty about diagnosis; probably not CHF or not CHF.

### Analyses

Stata 8.0 was used for the statistics. Cohen's kappa test (κ) was used to determine interrater agreement regarding the coding of the think-aloud protocols and the classification of the diagnostic judgements. Kappa values are classified as follows: <0, worse than chance; 0 to 0.2, poor; 0.21 to 0.4, fair; 0.41 to 0.6, moderate; 0.61 to 0.8, good; and >0.8, very good [22].

The research ethics committee of Huddinge University Hospital approved the study.

## Results

### Reliability – interrater coding agreement

#### Think-aloud protocols

The randomly selected test protocols contained 322 segments of propositions, 36 of which were excluded since they contained variables that were not going to be investigated in this study (e.g. treatment suggestions). There was disagreement between the two coders about the content of variables in 14 of the remaining 286 segments (4.8%). The remaining 272 segments were then tested for interrater agreement on argument values (for CHF; against CHF; just a mention), which was 95% (κ 0.85).

#### Diagnostic judgements

The interrater agreement was 92% (κ 0.85). For the few diagnostic judgements where there was initial disagreement, it was possible to agree upon an interpretation.

### Diagnostic reasoning

#### Assessments to be performed routinely according to guidelines

**Figure 1:**
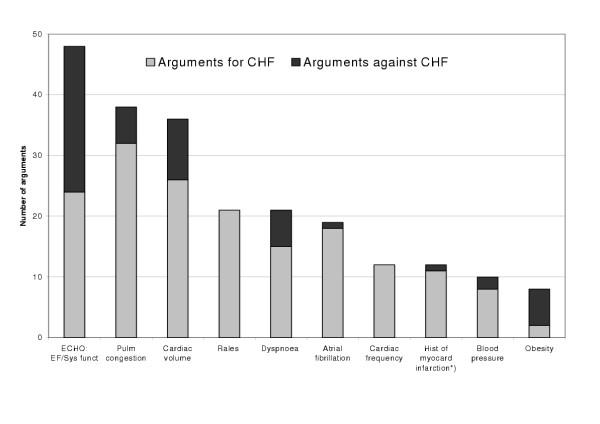
**The most frequently used diagnostic arguments **The ten most frequently used arguments making use of different categories of clinical information. Number of arguments favouring the diagnosis CHF and number of arguments against CHF are given for each category. One variable (indicated by *) was only presented in five of the vignettes.

The information that was used most frequently in diagnostic arguments was the ejection fraction value on ECHO, pulmonary congestion, and cardiac volume (Figure 1). The most frequent argument for CHF was pulmonary congestion on chest X-ray, and the most frequent argument against CHF was the ejection fraction value.

Symptoms and signs were not often used as arguments in the GPs' diagnostic reasoning (Figure 1). Symptoms were most frequently used as diagnostic arguments when reasoning about CV2 (Additional file 1), which represented the prototypical CHF case, with dyspnoea when walking on level ground and orthopnea ("in need of three pillows to be able to sleep"), and about CV5, which represented the prototypical non-CHF case (absence of dyspnoea). Signs were most frequently used for two of the case vignettes. In CV1, the presence of rales was used by nine of the GPs as an arguments for CHF (eight of them incorrectly ending up with this as the final diagnosis), and in CV3, tachycardia was used by nine of the GPs as an argument for CHF (eight of them correctly ending up with this as the final diagnosis). CV3 also had a gallop rhythm, which is reported to be fairly specific for CHF. However, only one GP used this as an argument for CHF.

According to the guidelines, a normal ECG opposes the diagnosis of CHF (Table 1). Only one of the patients (CV5) had a normal ECG. Three of the GPs used this information as an argument against CHF when reasoning about this patient, and two of them diagnosed the patient as not CHF. Four GPs used a pathological ECG as an argument for CHF (CV1, CV6), and all four diagnosed those patients as CHF.

According to the guidelines, information about cardiac enlargement or pulmonary congestion on chest X-ray gives some support for CHF if there are any pathological findings, and is of some importance as an argument against CHF if the findings are normal (Table 1). Chest X-ray findings were frequently used by the GPs as arguments in their diagnostic reasoning (Figure 1). When considered separately, information about cardiac volume was used as an argument 36 times (26 for, and 10 against CHF), and information about pulmonary congestion 38 times (32 for, and 6 against CHF).

#### ECHO findings as arguments for or against CHF

Each of the 15 GPs judged 6 case vignettes, which resulted in 90 judgement situations. In 48 of them, the EF value (or the information about left ventricular function in CV3) was used as an argument for or against CHF (Table 3). In some of the judgement situations in which the EF value was not used as an argument, other ECHO information was utilized, such as left ventricular hypertrophy or restricted motility of the ventricular wall. However, in 33 judgment situations, no ECHO information was used as an argument in the diagnostic reasoning. Table 3 shows the use of ECHO in all the judgement situations: Five GPs used information about EF in their diagnostic reasoning for five of the case vignettes, four GPs used it for four of the vignettes, one GP used it for three of the vignettes, one GP for two of the vignettes and two GPs for one of the vignettes (and in one case in the wrong direction). Two GPs never used information about EF in their diagnostic reasoning. Some of the GPs expressed uncertainty about the EF values (e.g. GP14, CV1: "I think... think I am not certain about the meaning of ejection fraction").

**Table 3:** GPs' use of ECHO information

	CV2	CV6	CV3	CV5	CV4	CV1
	CHF	CHF	CHF	Not CHF	Not CHF	Not CHF
GP1	**+**	other	other	0	0	0
GP2	+	+	0	0	0	other
GP3	+	+	0	-	-	-
GP4	+	+	0	-	-	0
GP5	+	+	other	-	-	-
GP6	+	+	0	-	-	-
GP7	+	+	0	-	-	other
GP8	+	+	0	-	-	0
GP9	+	+	0	-	0	-
GP10	+	+	-	**-**	-	0
GP11	+	+	0	-	-	-
GP12	0	0	other	0	other	other
GP13	+	+	0	0	0	-
GP14	0	0	other	0	0	0
GP15	0	0	0	0	+	0

Use of information about ejection fraction (EF) value, or, in the case of CV3, about left ventricular function, as arguments for (+) or against (-) the diagnosis CHF. "Other" indicates that other ECHO information than the ejection fraction was used in the diagnostic reasoning and (0) that no ECHO informtion was used. (CHF = chronic heart failure, CV = case vignette, GP = general practitioner)

In 17 judgement situations, there was a conflict between the GPs' evaluations of the chest X-ray information and their evaluations of the ECHO information. In seven of those situations, the final diagnosis was in the same direction as the ejection fraction argument (four CHF, three not CHF). In three judgement situations, the final diagnosis was in the same direction as the X-ray argument (three CHF). In seven judgement situations, the GP was uncertain about the diagnosis.

#### Other relevant diseases

In our study, the GPs used other diseases as an argument in a total of 70 judgement situations, mostly as arguments for CHF (91%). Atrial fibrillation, emphysema, history of myocardial infarction, and hypertension were the diagnoses most commonly used in this way.

#### Information that GPs disagree about

For certain variables, the same information value was used by some GPs as an argument for and by others as an argument against CHF. The presence of emphysema was sometimes seen as increasing the risk of CHF (e.g. GP 7, CV3: "...and then she has emphysema ... chronic obstructive pulmonary disease, which can also contribute to CHF."), or as an alternative explanation for symptoms (e.g. GP 13, CV3: "And then she also has emphysema, which could give her this severe breathlessness.").

Diabetes could also be seen as increasing the risk of CHF (e.g. GP 9, CV5: "...if I think the patient has CHF? Well, there are some facts in particular, she's diabetic, and she has uncontrolled hypertension, well, too high, and stasis – so I couldn't rule out the idea of CHF after all."), or as an alternative explanation for symptoms (e.g. GP 1, CV5: "... we have to improve her diabetes, since her fatigue may be due to that.").

Advanced age could be seen as increasing the probability of CHF (e.g. GP 2, CV6: "He's the age for it!"), or as an alternative explanation for symptoms (e.g. GP 1, CV6: "I'm not so sure that CHF alone can explain his symptoms. After all, he's 84 years old."). Age was used as a diagnostic argument only for the two patients over 80 years of age.

For relative cardiac volume, the reasoning could be compatible with GPs using different threshold values in their reasoning. The two lowest values were only used as arguments against CHF, the two highest values only as arguments for it, and the two intermediate values were used as arguments in both directions.

### Diagnostic judgements

There was total agreement among the GPs only for the prototypical CHF case; otherwise there was a large variation among GPs regarding diagnoses. Case vignettes representing CHF patients were more likely to be correctly diagnosed than those representing non-CHF patients (Table 4).

**Table 4:** GPs' classifications of case vignettes

	CV2	CV6	CV3	CV5	CV4	CV1
	CHF	CHF	CHF	Not CHF	Not CHF	Not CHF
Total number of arguments used by the GPs (proportion of arguments for CHF)	60 (98%)	52 (75%)	54 (85%)	57 (44%)	63 (70%)	63 (75%)
Number of GPs classifying the patient as CHF	**15**	**11**	**11**	3	6	11
Uncertain, no classification	0	2	2	3	3	1
Correct diagnoses of CHF cases and not CHF cases (proportion of correct diagnoses)	37/45 (82%)			18/45 (40%)		
Correct diagnoses, all judgements (proportion of correct diagnoses)	55/90 (61%)					

The GPs' classification of the case vignettes and the number of arguments used by the group of GPs. Cells with bold numbers indicate correct judgements. (CV = case vignette, CHF = chronic heart failure)

## Discussion

### GPs' diagnostic reasoning compared with guidelines

When comparing the GPs' diagnostic reasoning with guidelines, we found that the clinical information in the case vignettes was not used to the extent recommended in the guidelines. It is true that information about the ejection fraction value on ECHO was the single most frequent diagnostic argument, and it was the most common argument against CHF. This is in line with the guidelines, which emphasize the need for objective evidence of cardiac dysfunction. However, in more than one third of the judgement situations, the information about ECHO that was presented was not used as an argument. Over-diagnosis of CHF in primary health care has been demonstrated in a number of studies, with ECHO findings as the gold standard [3,4,15]. Limited access to ECHO has been suggested as an explanation for this finding. However, our data indicate that simply providing access to ECHO might not be enough.

In the diagnostic algorithm, symptoms and signs are the entry criteria. However, the GPs did not seem to use them consistently in this way, except when diagnosing the prototypical CHF and non-CHF cases. One reason for this might be that most symptoms and signs considered typical for CHF are fairly non-specific as regards the diagnosis CHF.

Information about other relevant diseases, which was important in the GPs' diagnostic reasoning, is not included in the list of assessments to be performed routinely (Table 1) [9]. However, information about a history of myocardial infarction, for example, increases the probability of CHF. In a study of CHF diagnostics in primary health care, it was shown that the combination of cardiac enlargement and a history of myocardial infarction had the best positive predictive value for CHF when systolic dysfunction measured by ECHO was used as gold standard [23]. This finding is compatible with the notion that experienced physicians structure their knowledge more according to enabling conditions than according to biomedical reasoning [24-26]. Enabling conditions are patient contextual factors such as sex, age, medical history, and occupation. In most routine diagnostic situations, biomedical details of a disease and its cause are not so important, and the physician's images of the diseases ('illness scripts') are rather characterized by these enabling conditions, which form a characteristic pattern. The GPs' frequent use of this kind of information may thus indicate that they are experienced physicians, with illness scripts for CHF which include other diseases. It might be valuable to include this kind of information in a clearer way in the guidelines, because it would reflect the higher probability of CHF in patients with those characteristics.

### Some methodological considerations

The case vignettes represented authentic patients referred by GPs to a cardiology department for problems related to heart failure. This may have led to a selection of more complicated patients than the "typical" heart failure patients in primary health care. The reason we chose this group of patients was that we wanted to include patients who were thoroughly investigated, with a well-founded clinical diagnosis, and for whom information about all variables of interest could be found in the patient records. Selecting GPs only from health care centres in, or relatively close to, the city centre may have biased the results, since differences in catchment areas, working conditions, and access to echocardiography may influence GPs' diagnostic habits. This could make it difficult to generalize the results to other GPs. Only 50% of the GPs who were contacted agreed to take part in the study, which could bias the results. However, since the age distribution was the same in the two groups, it seems unlikely that the drop-out group would differ from the study group regarding clinical experience.

### Guidelines as decision support when diagnosing CHF

The full version of the guidelines is difficult to apply to individual diagnostic situations and it is also difficult to use it for assessment of diagnostic behaviour [13]. In this study, we have used the table of routine assessments as a reference for evaluating the GPs' diagnostic reasoning (Table 1). This table includes a rough weighting of the importance of different types of information, which could serve as a guide for diagnostic judgements, even if it is not obvious how it should be used in individual cases. The two compulsory criteria in the definition are included in this table as necessary conditions. However, in some situations these judgment tools will not be satisfactory. One example is case vignette CV3, where the clinical picture was strongly indicative of CHF, with dyspnoea, rales, tachycardia, gallop rhythm, cardiomegaly and pulmonary congestion, while according to ECHO findings there was normal left ventricular function (Additional file 1). The patient could therefore be classified as a non-heart failure patient according to the definition, while the clinical diagnosis, based on the attending cardiologist's judgement of all accessible information, was in fact heart failure. However, the ECHO in this case also included information about atrial dilatation, mitral insufficiency and pulmonary hypertension, i.e. a rather complex situation. A patient with clinical findings suggestive of CHF, but with a normal ejection fraction value, could be considered not to have CHF, i.e. not to have a systolic CHF, but could alternatively have a diastolic CHF [27,28]. This situation is not dealt with in the guidelines.

### Some implications of this study

GPs' tendency to over-diagnose CHF has been explained by their relying on symptoms, signs and less specific investigations such as chest X-ray, and by limited access to ECHO in the primary health care. However, this study indicates that a substantial minority of GPs seem to be less familiar with the use of ECHO and EF. Thus, access to ECHO ought to be accompanied by education about how to integrate this information better in the diagnostic reasoning.

Guidelines ought to include search of information about other cardio-vascular diseases in the list of assessments to be performed routinely (Table 1) and in the algorithm for diagnosis of heart failure. This would reflect the increased probability of CHF in presence of those diseases. The problem of diastolic heart failure should also be addressed in a clearer way in guidelines.

## Conclusions

The information in the case vignettes was underused as arguments for and against the possibility of CHF as compared with the guidelines. Information about the EF value was the single most frequently used argument for or against CHF; nevertheless, in one third of the diagnostic judgements the GPs did not consider any information about the ECHO in their diagnostic reasoning. Information about symptoms and signs were not used to to the extent suggested in the guidelines. Information about other relevant diseases was frequently used in the GPs' diagnostic reasoning, indicating that they often relied on illness scripts. Some implications of our study are that 1) GPs should be taught how to use ECHO information better in their diagnostic reasoning, 2) guidelines ought to give more importance to information about other cardio-vascular diseases in the diagnostic reasoning, and 3) guidelines ought to treat the topic of diastolic heart failure in a clearer way.

## List of abbreviations used

CHF Chronic heart failure

COPD Chronic obstructive pulmonary disease

ECHO Echocardiography

ECG Electrocardiography

EF Ejection fraction

GP General practitioner

## Competing interests

The author(s) declare that they have no competing interests.

## Authors' contributions

All authors conceived of the study and participated in the design. YS carried out the data collection, performed the statistical analyses and drafted the manuscript. All authors participated in the interpretation of the results and the discussions of the drafts. All authors read and approved the final manuscript.

## Pre-publication history

The pre-publication history for this paper can be accessed here:



## Supplementary Material

Additional File 1 **Case vignette characteristics **The additional file shows some important characteristics of the case vignettes. Format: Word-table. Click here for file
