# Supplementary material for: General practitioners' reasoning when considering the diagnosis heart failure: a think-aloud study
Source: BMC Fam Pract. 2005 Jan 15;6:4. doi: 10.1186/1471-2296-6-4 (PMC546006; doi:10.1186/1471-2296-6-4)
Supplement: Additional File 1 — Case vignette characteristics The additional file shows some important characteristics of the case vignettes. Format: Word-table. [file 1471-2296-6-4-S1.doc]

# Additional files

## **Additional file 1 – Case vignette characteristics**

Case vignettes: Important characteristics. Case vignette numbers represent the order of presentation. (AF = atrial fibrillation, CHF = chronic heart failure, CV = case vignette, EF = ejection fraction, ECG = electrocardiography, ECHO = echocardiography, LV = left ventricle, MI = mitral valve insufficiency).

|  | CV2  Man, 70, CHF | CV6  Man, 84, CHF | CV3  Woman, 72, CHF | CV5  Woman, 73, not CHF | CV4  Man, 81, not CHF | CV1  Man, 64, not CHF |
| --- | --- | --- | --- | --- | --- | --- |
| Appropriate symptoms | Dyspnoea when walking on level ground, orthopnea | Fatigue, dyspnoea when walking on level ground | Dyspnoea when walking on level ground | Fatigue, leg oedema | Dyspnoea on climbing | Fatigue, dyspnoea when walking on level ground |
| Appropriate signs | Rales, dyspnoea at rest | Rales | Rales, tachycardia, gallop rhythm | Rales, leg oedema | Rales | Rales |
| ECG | AF, freq 90/min, right bundle block | Sinus rhythm, freq 70/min, pathol R-progression anterior | AF, freq 130/min | Sinus rhythm, freq 80/min, no Q-waves | Sinus rhythm,  freq 74/min,  ST-depression | AF, freq 75/min, ST-depression |
| X-ray: Relative cardiac volume (ml/ml^2^) | 920 | 470 | 820 | 460 | 630 | 520 |
| X-ray: Pulmonary congestion | Congestion | No congestion | Congestion | Congestion | Congestion | Congestion |
| ECHO: EF or LV function | EF 25% | EF 35% | LV function normal, MI | EF 55% | EF 55% | EF 55% |
